# Family functioning in students of health sciences in four Latin American countries: a study of the structure and factorial invariance of the FACES III scale. A cross-sectional study

**DOI:** 10.1186/s41155-024-00287-1

**Published:** 2024-02-05

**Authors:** Lindsey W. Vilca, Víctor Díaz-Narváez, Aracelis Calzadilla-Núñez, Claudia Arispe-Alburqueque, Susana Facio Arciniega, María Alejandra Orostegui, Herminia Castellón-Montenegro, Karina Santander, Claudio López-Labarca, Guiomar Hernández Álvarez, Shirley Fernández-Aragón, Luz Marina Alonso Palacio, Alejandro Reyes-Reyes, Marco Cervantes Mendoza

**Affiliations:** 1 South American Center for Education and Research in Public Health, Universidad Norbert Wiener, Lima, Peru; 2 Departamento de Investigaciones, Universidad Andres Bello, Santiago, Chile; 3 Departamento de Psiquiatría, Universidad Bernardo OHiggins, Santiago, Chile; 4 Departamento de Investigaciones, Universidad Norbert Wiener, Lima, Peru; 5 Universidad Autónoma de Coahuila (Unidad Torreón), Facultad de Enfermería, Coahuila, Mexico; 6 Departamento de Enfermería, Universidad Simón Bolívar, Barranquilla, Colombia; 7 Universidad Metropolitana, Facultad de Enfermería, Barranquilla, Colombia; 8 Departamento de Enfermería, Universidad de Atacama, Copiapó, Chile; 9 Departamento de Obstetricia y Puericultura, Universidad de Atacama, Copiapó, Chile; 10 Universidad de Cartagena, Facultad de Enfermería, Cartagena, de Indias, Colombia; 11 Departamento de Enfermería, Corporación Universitaria Rafael Núñez, Cartagena, de Indias, Colombia; 12 Universidad del Norte, División de Ciencias de la Salud, Barranquilla, Colombia; 13 Departamento de Psicología, Universidad Santo Tomás, Facultad de Ciencias Sociales y Comunicaciones, Concepción, Chile; 14 División Humanidades y Ciencias Sociales, Departamento de Psicología, Universidad del Norte, Barranquilla, Colombia

**Keywords:** Family functioning, Cohesion, Adaptability, FACES III, Factorial Invariance, Confirmatory factor analysis

## Abstract

**Background:**

Psychometric studies of the FACES III scale in Spanish-speaking countries show a lack of agreement on the factorial structure of the scale. In addition, most of the studies have only performed exploratory analyses of its factorial structure.

**Objective:**

The objective of the present study was to confirm the structure and factorial invariance of the FACES III scale in nursing and obstetric students from Chile, Colombia, Peru, and Mexico.

**Methods:**

A total of 3303 students from the four countries participated in this study (Colombia = 1559, Chile = 1224, Peru = 215, Mexico = 305).

**Results:**

The results of the study showed that the Bi-factor model presents the best-fit indexes to the data from Colombia, Chile, and Mexico, but not from Peru. In addition, it was found that this model showed evidence of being strictly invariant among the three countries in the sequence of the invariance models proposed: metric invariance (ΔRMSEA = .000), scalar (ΔRMSEA = .008), and strict (ΔRMSEA = .008). The bi-factor model also showed adequate reliability indexes in the three countries.

**Conclusion:**

It is concluded that the FACES III scale shows adequate psychometric performance under a bi-factor model in nursing and obstetric students from Colombia, Chile, and Mexico. The lack of fit of the model in Peru could be associated with the small sample size.

## Introduction

Recent studies have shown that poor family functioning is associated with the presence of non-suicidal self-injury (Wang et al., 2022), depression (Freed et al., 2016), aggressive conduct (Pérez-Fuentes et al., 2019), decreased levels of life satisfaction (Szcześniak & Tułecka, 2020), and mental health problems (Scully et al., 2019). In this context, several studies have shown the role of family functioning in health sciences students during the Covid-19 pandemic. A study performed in China on medical students found that adequate family functioning is related to a lower presence of depressive and anxiety symptoms (Shao et al., 2020). Similarly, another study performed in the same country on medical, nursing, and medical technology students reported that good family functioning is associated with a decrease in the risk of distress and stress (Li et al., 2020). Another study conducted in the USA on nursing students showed that better family functioning is related to lower stress, anxiety, and depression (Kim et al., 2021). In Nigeria, a study of health science students found that negative family functioning is associated with a higher level of depression (Ojewale, 2021).

Several theoretical models that explain family functioning can be found in the scientific literature, such as the family systems model (Beavers & Hampson, 2000), the McMaster model of family functioning (Miller et al., 2000), and the family process (Skinner et al., 2000). However, the circumplex model of the marital and family system is one of the most widely used models to explain family functioning (Olson et al., 2000, 2019). Under this model, family functioning is the capacity of the family system to satisfy the needs for affection, care, socialization, and family status, following the norms of the society to which it belongs (Dickinson Bannack et al., 1998). This model states that family functionality is made up of two components: cohesion and adaptability. Family cohesion refers to the emotional bond between the members of the system and the internal and external boundaries of the family. Family adaptability is defined as the capacity of the family system to change its power structure, roles, and relationship patterns in response to situational or evolutionary stress (Olson et al., 2019).

Additionally, a third component was added, communication, which plays a facilitating role in developing and improving the first two components (Olson et al., 2000). The three components were discovered by pooling concepts developed in couples and family therapy (Olson et al., 1979, 2019). It is important to mention that Cohesion and Adaptability are not linear concepts but curvilinear; very high or very low levels show dysfunction. For each dimension, three central levels are considered balanced, and the extreme levels, whether low or high, are considered unbalanced. The union of both components results in nine balanced systems (balanced in both dimensions), twelve midrange systems (balanced in only one), and four unbalanced systems (unbalanced in both dimensions) (Olson et al., 2019). Three major hypotheses are derived from this model: (a) families and couples with balanced systems will function better throughout the family life cycle than unbalanced systems; (b) families and couples with balanced systems have more positive communication than unbalanced systems; and (c) families and couples with balanced systems will better cope with stressful situations and changes throughout the family life cycle. It is important to mention that the circumplex model is dynamic; that is, families can modify their levels of Cohesion and Adaptability in order to improve their family functioning (Olson et al., 2019). Several studies have shown that the circumplex model is relevant to explain behavior problems (Joh et al., 2013), drug use (Tafà & Baiocco, 2009), suicidal ideation (Ortiz-Sánchez et al., 2023), self-control (Gomes & Gouveia-Pereira, 2020), among other constructs.

The Family Adaptability and Cohesion Evaluation Scale (FACES) has been developed based on this theoretical model. It presents several versions; among them, the most used is the FACES III scale since it allows a curvilinear evaluation of family functioning. This version has twenty items distributed in two dimensions: Adaptability and Cohesion. Both dimensions present four levels that, when combined, identify sixteen types of families (Olson, 1986). More than 1200 studies on the circumplex model have been developed using the FACES III scale (Olson et al., 2019). In addition, in the context of mental health, one of the most widely used instruments to study family functioning is the FACES III scale (Souza et al., 2011). Also, this version has been used to study the relationship between the dimensions of cohesion and adaptability with other variables such as anxiety, depression, quality of family life, emotional expression, and psychological discomfort (Koutra et al., 2016; X. Lei & Kantor, 2022; Park et al., 2018). Everything shown above evidences the theoretical and practical importance of this instrument.

On the other hand, although there is a new version of the scale called FACES IV, it is not as widely used as FACES III in the studies carried out in Ibero-America. This could be due on the one hand to the length of the test, sixty-two questions as opposed to twenty questions in FACES III, and on the other hand, it could be because FACES IV require a fee for its use. Regarding the psychometric performance of the FACES III scale, several studies conducted in Latin America have explored the factor structure of the scale. In Argentina, a confirmatory factor analysis showed that the original two-factor model is not entirely adequate and that a three-factor model with three related factors is a better fit for the data (Schmidt et al., 2010). In Mexico, an exploratory factor analysis showed that a two-factor related model is possible if items in both factors, especially in adaptability, are eliminated (Ponce Rosas et al., 2002). Similarly in Chile, an exploratory factor analysis showed that the items form three related factors, where the total variance explained (43%), belongs mostly to the first factor (Zicavo et al., 2012). Another study conducted in the same country found that the items fit a second-order factor model with seven specific factors (Zegers et al., 2003). In Peru, a confirmatory factor analysis showed that a two-factor related model fits the data (Bazo-Álvarez et al., 2016). However, another study carried out in the same country showed that only the cohesion dimension presented adequate adjustment indexes and that the family adaptation dimension did not show an adequate internal structure (Villarreal-Zegarra & Paz-Jesús, 2017). It is important to note that no other psychometric studies were found in the countries mentioned above or in any other Latin American country that analyzed the internal structure of the FACES III scale.

As can be seen in the previous studies, there is no agreement on the factor structure of the scale. In addition, most studies in the countries mentioned above have conducted only exploratory studies and therefore the factor structure of the FACES III scale cannot be confirmed. Confirming the factorial structure of the scale is essential since it guarantees a reliable and valid measurement of family functioning; that is, it guarantees that the dimensions proposed in the theoretical model are being measured. In addition, it allows adequate management of the scores derived from the scale (Brown, 2015). On the other hand, no cross-cultural studies have been found that study the factor invariance of the scale in Ibero-American countries. This evidence is important as it is a prerequisite for comparative studies (Rutkowski & Svetina, 2017). The lack of factorial invariance would not make it possible to ensure that the differences found between the different groups are real differences in the construct and that, on the contrary, these differences could be associated with the psychometric characteristics of the scale (Guenole & Brown, 2014). Among the most common causes for the lack of invariance are differences in the interpretation of the construct between the groups, differences in the interpretation of the items, the cultural context when answering the items, or the presence of items that work better in a group but not in the other (Shi et al., 2019). On the other hand, guaranteeing the factorial invariance of the scale would make it possible to carry out comparative studies on family functioning between countries, allowing a better understanding of this construct in different cultural contexts.

Therefore, the objectives of this study are as follows: (a) to study the validity based on the internal structure of the FACES III scale in the countries of Colombia, Chile, Peru, and Mexico; (b) to evaluate the factor invariance of the FACES III scale in these countries; and (c) to estimate the degree of reliability of the FACES III scale in these countries.

## Method

### Participants

The study included 3303 nursing and obstetric students from universities in Colombia (Universidad Simón Bolívar, Universidad Metropolitana, Universidad de Cartagena, and Corporación Universitaria Rafael Núñez), Chile (Universidad de Atacama and Universidad San Sebastián), Peru (Universidad Norbert Wiener), and Mexico (Universidad de Coahuila). Table 1 shows that the average age of participants living in Colombia is 21.9 years old (*SD* = 3.8). A similar pattern is observed for participants living in Chile (*M* = 22.1; *SD* = 3.2), Peru (*M* = 22.3; *SD* = 3.8), and Mexico (*M* = 20.6; *SD* = 3.4). In addition, in all countries, there is a higher proportion of women (Colombia = 63.4%; Chile = 83.1%; Peru = 97.7%; Mexico = 78%) than men (Colombia = 36.6%; Chile = 16.9%; Peru = 2.3%; Mexico = 22%). Finally, all participants in Colombia and Mexico are studying nursing, in contrast to Chile where 84.2% are studying nursing and 15.8% are studying obstetrics. In Peru, all participants are studying obstetrics.

**Table 1:** Sociodemographic characteristics of the participants

Sociodemographic data	Colombia (*n* = 1559)	Chile (*n* = 1224)	Peru (*n* = 215)	Mexico (*n* = 305)
Age (M ± SD)	21.9 ± 3.8	22.1 ± 3.2	22.3 ± 3.8	20.6 ± 3.4
Sex, *n* (%)				
Male	570 (36.6%)	207 (16.9%)	5 (2.3%)	67 (22%)
Female	989 (63.4%)	1017 (83.1%)	210 (97.7%)	238 (78%)
Studies, *n* (%)				
Nursing	1559 (100%)	1030 (84.2%)	0 (0%)	305 (100%)
Obstetrics	0 (0%)	194 (15.8%)	215 (100%)	0 (0%)

*M* mean, *SD* standard deviation

### Instruments

#### Family Adaptability and Cohesion Evaluation Scale (FACES III)

For the study, we used the version adapted to Spanish by Zicavo et al. (2012), consisting of 20 items that measured two dimensions: cohesion (1, 4, 5, 8, 10, 11, 13, 15, 17 y 19) and adaptability (2, 3, 6, 7, 9, 12, 14, 16, 18, 20). In addition, the items present five response categories that are scored as follows: Never (0), Seldom (1), Sometimes (2), Often (3), and Almost always (4).

The scale was initially developed in the USA (Olson, 1986). This version is an improvement over previous versions (FACES I and FACES II) since Cohesion and Adaptability are measured curvilinearly. FACES III is usually used in family therapy and research contexts (Olson et al., 2019).

#### Procedure

For the study, approval was obtained from the ethics committee of the Universidad de San Sebastián, Chile (Final Resolution N^o^. 83/ 2020/02), and the standards established in the Declaration of Helsinki were followed (World Medical Association, 2013). The data were obtained in July 2020 and in all countries, the collection process was the same and is subject to the principle of confidentiality.

A non-probabilistic convenience sampling method was used for data collection and a virtual form was applied in the classrooms. In the online form, the informed consent, the objectives of the study, and the contact information of the study coordinators were presented first. Only after providing informed consent, students accessed the FACES III scale items. During the data collection process, the confidentiality of the data and the possibility of withdrawing from the evaluation at any time were guaranteed.

#### Data analysis

For the confirmatory factor analysis (CFA), the robust maximum likelihood estimator (MLR) was used (Yuan & Bentler, 2000). Root mean square error of approximation (RMSEA), standardized root mean square residual (SRMR), Comparative Fit Index (CFI), and Tucker-Lewis Index (TLI) indices were used to evaluate the fit of the models. For the RMSEA and SRMR indices, values less than .08 were considered acceptable (Kline, 2016). For the CFI and TLI indices, values greater than .95 were considered adequate (Schumacker & Lomax, 2015). The omega coefficient was used to evaluate the scale’s reliability (McDowell, 2006), where a value greater than .70 is adequate (Viladrich et al., 2017). The H coefficient was also used to evaluate how well a latent variable is represented by a set of items (Mueller & Hancock, 2001). For the Bi-factor models, the hierarchical omega coefficient was used (Zinbarg et al., 2005). The explained common variance was reported to evaluate the strength of the general factor in the Bi-factor models (Sijtsma, 2009).

Multi-group confirmatory factor analysis (MGCFA) was used to evaluate the factor invariance of the scale according to the nationality of the participants (country), where a sequence of hierarchical invariance models was proposed. First, configural invariance (reference model) was evaluated, followed by metric invariance (equality of factor loadings), scalar invariance (equality of factor loading and intercept), and finally, strict invariance (equality of factor loadings, intercept, and residuals). A formal statistical test was first used to compare the sequence of models, for which the chi-square difference (Δχ2) was used, where non-significant values (*p* > .05) suggest invariance between groups. Second, a modeling strategy was used, for which the differences in the RMSEA (ΔRMSEA) were used, where differences less than < .015 show the invariance of the model between the groups (Chen, 1997).

All statistical analyzes were performed using the “lavaan” package (Rosseel, 2012) for the CFA and the “semTools” package (Jorgensen et al., 2018) for factor invariance. The RStudio environment (RStudio Team, 2018) for R (R Core Team, 2019) was used in all cases.

#### Ethical approval

All procedures performed in the study were approved by the university’s ethics committee and conformed to the requirements of the 1975 Declaration of Helsinki. Informed consent was obtained from all participants included in the study.

## Results

### Descriptive analysis

Table 2 shows that item 13 (“Family members support each other in difficult times”) has the highest average score in the four countries. Meaning, most of the participants indicate that this behavior occurs frequently in the family experience. It can also be seen that item 18 (“Parents and children talk about punishments and rules”) presents the lowest average score in all countries, i.e., most of the participants indicate that this behavior rarely occurs in the family experience. In addition, the response rate of participants in all countries was similar. Regarding the asymmetry (*g1*) and kurtosis (*g2*) indices, all the items present adequate indices (*g1* < ± 2; *g2* < ± 7), according to the criteria of Finney and DiStefano (2013).

**Table 2:** Item descriptive analysis and item response rates

Country	Items	*M*	*SD*	*g1*	*g2*	Response rate				
						0	1	2	3	4
Colombia (*n* = 1559)	1	3.11	1.12	− 1.11	.19	2.6%	10.3%	11%	25.7%	50.5%
	2	2.77	1.15	− .70	− .39	4.4%	11.9%	18.3%	33.4%	32%
	3	3.06	1.06	− 1.03	.25	2.2%	9%	12.7%	32.1%	43.9%
	4	2.96	1.10	− .90	− .02	3.1%	9.6%	15.5%	32.2%	39.6%
	5	3.21	1.00	− 1.23	.73	1.4%	7.8%	10.1%	29.8%	50.8%
	6	2.86	1.13	− .81	− .21	4%	10.4%	17.2%	32.5%	35.9%
	7	2.99	1.04	− .94	.16	2.2%	9.5%	13.5%	36.9%	37.9%
	8	2.80	1.15	− .71	− .44	3.8%	12.6%	17.3%	32.4%	33.9%
	9	3.12	1.09	− 1.15	.36	2.4%	10%	9.7%	28.4%	49.4%
	10	2.88	1.17	− .81	− .33	4%	11.2%	16.6%	28.7%	39.4%
	11	2.83	1.13	− .71	− .43	3.2%	12.3%	17.6%	32.3%	34.6%
	12	2.87	1.11	− .75	− .34	3%	11.2%	17.8%	31.7%	36.3%
	13	3.37	.94	− 1.55	1.75	1.1%	5.8%	8.1%	24.8%	60.2%
	14	3.00	1.05	− .91	.04	2%	9.7%	14.3%	34.8%	39.2%
	15	2.91	1.07	− .86	.72	3.1%	8.9%	17%	36.4%	34.6%
	16	2.89	1.08	− .81	− .14	2.7%	10.7%	16%	36.1%	34.4%
	17	2.58	1.17	− .48	− .73	4.9%	16%	20.7%	33%	25.5%
	18	2.66	1.22	− .64	− .55	6.9%	11.7%	20.5%	30.5%	30.5%
	19	2.90	1.21	− .92	− .21	5.4%	11.2%	12.8%	29.2%	41.5%
	20	2.79	1.16	− .74	− .42	4.4%	12.9%	15.8%	33.7%	33.2%
Chile (*n* = 1224)	1	3.42	.77	− 1.34	1.67	.3%	2.1%	9%	31.9%	56.7%
	2	3.05	.96	− 1.02	.79	2.1%	5.1%	15.7%	39.4%	37.7%
	3	3.13	.94	− 1.18	1.28	2.1%	4.2%	13.2%	39.3%	41.1%
	4	3.05	.98	− 1.06	.89	2.6%	4.7%	15.9%	38.9%	37.8%
	5	3.37	.86	− 1.39	1.65	.8%	3.2%	10.8%	29%	56.2%
	6	2.90	1.05	− .98	.59	4.4%	5.3%	18.5%	39.3%	32.4%
	7	3.06	.94	− 1.01	.83	1.7%	5.5%	14.5%	42%	36.3%
	8	2.78	1.12	− .57	− .58	3.2%	11%	24.3%	27.6%	33.9%
	9	3.57	.76	− 2.05	4.56	.7%	2%	6%	22.5%	68.9%
	10	3.35	.93	− 1.47	1.63	1.3%	4.7%	10.5%	25.1%	58.5%
	11	2.76	1.01	− .61	− .12	2.5%	8.8%	24.3%	38.7%	25.7%
	12	3.06	1.01	− 1.02	.58	2.6%	5.1%	17.3%	33.4%	41.5%
	13	3.71	.62	− 2.29	5.56	.2%	.8%	5.1%	16.3%	77.7%
	14	2.90	1.01	− .78	.18	2.5%	6.5%	21.3%	37.7%	31.9%
	15	2.74	1.06	− .63	− .17	3.5%	9.2%	24.2%	36.6%	26.6%
	16	3.08	1.02	− 1.12	.78	2.9%	5.4%	15%	33.8%	42.9%
	17	2.75	1.07	− .61	− .31	3.1%	10.2%	23.2%	35.2%	28.3%
	18	2.64	1.22	− .64	− .52	7.5%	10.9%	21.2%	31%	29.3%
	19	3.27	1.01	− 1.40	1.32	2.3%	5.3%	11.4%	25.3%	55.6%
	20	2.91	1.05	− .82	.06	2.9%	7.7%	19.6%	35%	34.8%
Peru (*n* = 215)	1	3.17	.89	− .86	.11	.5%	4.2%	17.2%	34.4%	43.7%
	2	2.73	.98	− .44	− .28	1.9%	7.9%	29.8%	35.8%	24.7%
	3	2.94	.99	− .83	.39	2.8%	4.2%	23.3%	35.3%	34.4%
	4	2.99	.93	− .51	− .54	.5%	5.1%	25.1%	33.5%	35.8%
	5	3.20	.87	− .89	.03	.9%	4.2%	14%	36.3%	44.7%
	6	2.81	.93	− .32	− .64	.5%	7.4%	29.3%	35.8%	27%
	7	2.80	.98	− .65	.23	2.8%	5.1%	27.4%	38.1%	26.5%
	8	2.61	.99	− .39	− .31	2.3%	10.7%	30.2%	37.2%	19.5%
	9	3.33	.80	− 1.21	1.46	.5%	2.8%	9.8%	37.7%	49.3%
	10	2.94	.99	− .78	.12	1.9%	7%	20%	37.7%	33.5%
	11	2.75	1.01	− .45	− .61	.9%	12.6%	22.8%	38.1%	25.6%
	12	2.86	.95	− .57	− .11	1.4%	6.5%	25.1%	38.6%	28.4%
	13	3.39	.81	− 1.45	2.36	.9%	1.9%	9.8%	32.6%	54.9%
	14	2.92	.89	− .66	.34	1.4%	4.2%	23.7%	42.8%	27.9%
	15	2.67	.98	− .54	.01	2.8%	8.4%	28.4%	40%	20.5%
	16	2.92	.95	− .52	− .39	.9%	6%	26%	34.4%	32.6%
	17	2.67	.97	− .39	− .25	1.9%	8.8%	30.7%	37.7%	20.9%
	18	2.59	1.02	− .44	− .23	3.3%	10.2%	30.7%	36.3%	19.5%
	19	2.88	1.10	− .91	.24	4.7%	6.5%	19.5%	34.4%	34.9%
	20	2.65	1.02	− .39	− .43	2.3%	10.7%	29.8%	34.4%	22.8%
Mexico (*n* = 305)	1	3.23	.96	− 1.29	1.31	2%	3.9%	13.4%	30.2%	50.5%
	2	2.81	1.02	− .75	.13	3%	8.2%	21%	40.7%	27.2%
	3	2.96	1.04	− .91	.27	2.6%	7.5%	17%	36.7%	36.1%
	4	2.83	1.14	− .84	− .05	4.9%	8.9%	18.4%	34.1%	33.8%
	5	3.28	.89	− 1.19	1.03	1%	3.3%	14.1%	30.2%	51.5%
	6	2.50	1.21	− .52	− .52	8.9%	9.5%	28.2%	29.5%	23.9%
	7	2.90	1.08	− .83	.05	3.3%	7.9%	19.7%	34.1%	35.1%
	8	2.63	1.20	− .59	− .48	7.2%	9.5%	25.6%	28.9%	28.9%
	9	3.40	.87	− 1.77	3.41	1.6%	3%	6.6%	31.1%	57.7%
	10	3.09	1.11	− 1.16	.53	3.6%	7.9%	12.1%	28.9%	47.5%
	11	2.58	1.09	− .62	− .09	6.2%	7.9%	28.5%	36.1%	21.3%
	12	2.90	1.09	− .81	.01	3.6%	6.9%	22%	30.8%	36.7%
	13	3.62	.67	− 1.92	3.95	.3%	.7%	6.9%	20.7%	71.5%
	14	2.85	1.05	− .82	.13	3.3%	8.5%	18.7%	39.3%	30.2%
	15	2.79	1.07	− .75	.00	3.9%	8.2%	22%	36.7%	29.2%
	16	2.89	1.13	− .87	− .09	3.9%	10.2%	15.7%	33.4%	36.7%
	17	2.73	1.18	− .74	− .27	6.6%	8.9%	21.3%	31.8%	31.5%
	18	2.44	1.26	− .51	− .70	10.8%	11.5%	23.6%	31.5%	22.6%
	19	2.93	1.12	− .98	.35	5.2%	4.9%	20%	30.8%	39%
	20	2.66	1.17	− .56	− .59	4.9%	13.4%	21.3%	31.8%	28.5%

*M* mean, *SD* standard deviation, *g1* Skewness, *g2* Kurtosis, 0 = Never, 1 = Seldom, 2 = Sometimes, 3 = Often, 4 = Almost Always

### Validity based on the internal structure

Table 3 shows the fit indexes of the models proposed for the family functioning scale in the scientific literature. It is observed that the original model with two related dimensions (model 1) does not show adequate fit indices in Colombia (RMSEA = .077 [CI 90% .073–.081]; CFI = .89; TLI = .88), Chile (RMSEA = .090 [CI 90% .086–.094]; CFI = .86; TLI = .85), Peru (RMSEA = .070 [CI 90% .058 – .081]; CFI = .88; TLI = .87) and Mexico (RMSEA = .088 [CI 90% .086–.094]; CFI = .88; TLI = .89). Similarly, a one-dimensional model (model 2) does not show adequate fit indexes in Colombia (RMSEA = .076 [CI 90% .072–.080]; CFI = .89; TLI = .88), Chile (RMSEA = .095 [CI 90% .090–.099]; CFI = .84; TLI = .83), Perú (RMSEA = .071 [CI 90% .060–.083]; CFI = .88; TLI = .86), and Mexico (RMSEA = .089 [CI 90% .080–.098]; CFI = .89; TLI = .88).

**Table 3:** Adjustment indexes of FACES III models in Colombia, Chile, Peru, and Mexico

Model	Country	χ^2^	df	*p*	CFI	TLI	SRMR	RMSEA [90% CI]	F1		F2		FG		
									ω	*H*	ω	*H*	ω_H_	*H*	*ECV*
1	Colombia	1214.58	169	< .001	.89	.88	.045	.077 [.073–.081]	.87^a^	.88	.87^a^	.88	–	–	–
	Chile	1412.85	169	< .001	.86	.85	.055	.090 [.086–.094]	.89^a^	.89	.89^a^	.90	–	–	–
	Peru	316.46	169	< .001	.88	.87	.058	.070 [.058–.081]	.84^a^	.86	.84^a^	.86	–	–	–
	Mexico	474.35	169	< .001	.88	.89	.050	.088 [.086–.094]	.90^a^	.91	.92^a^	.93	–	–	–
2	Colombia	1214.56	170	< .001	.89	.88	.045	.076 [.072–.080]	–	–	–	–	.93^a^	.93	–
	Chile	1550.13	170	< .001	.84	.83	.056	.095 [.090–.099]	–	–	–	–	.94^a^	.94	–
	Peru	326.01	170	< .001	.88	.86	.058	.071 [.060–.083]	–	–	–	–	.91^a^	.92	–
	Mexico	485.67	170	< .001	.89	.88	.050	.089 [.080–.098]	–	–	–	–	.95^a^		–
3	Colombia	1207.39	168	< .001	.89	.87	.045	.077 [.073–.081]	.87^c^	–	.87^c^	–	–	–	–
	Chile	1404.49	168	< .001	.86	.85	.055	.090 [.086–.095]	.89^c^	–	.89^c^	–	–	–	–
	Peru	314.58	168	< .001	.88	.87	.058	.070 [.058–.082]	.85^c^	–	.85^c^	–	–	–	–
	Mexico	471.54	168	< .001	.89	.88	.050	.088 [.078–.097]	.90	–	.91	–	–	–	–
4	Colombia	901.42	150	< .001	.92	.90	.038	.068 [.063–.072]	.08^b^	.35	.06^b^	.32	.91	.93	.90
	Chile	937.71	150	< .001	.92	.90	.039	.072 [.067–.076]	.17^b^	.62	.04^b^	.26	.88	.94	.84
	Peru^d^	–	–	–	–	–	–	–	–	–	–	–	–	–	–
	Mexico	347.55	150	< .001	.93	.92	.039	.073 [.063 – .083]	.13^b^	.45	.11^b^	.46	.88	.95	.88

χ2 Chi square, *df* degrees of freedom, *SRMR* standardized root mean square residual, *TLI* Tucker-Lewis Index, *CFI* comparative fit index, *RMSEA* root mean square error of approximation. Model 1 = Two correlated factor model, Model 2 = Unidimensional model, Model 3 = Second Order General Factor, Model 4 = Bi-factor model, a = Omega de McDonald, b = Hierarchical Omega, c = Compound reliability, H = Coefficient H, ωH = Hierarchical Omega, ECV = explained common variance, d = Convergence problems

Furthermore, a second-order general factor model (model 3) does not fit the data well in the countries studied: Colombia (RMSEA = .077 [CI 90% .073–.081]; CFI = .89; TLI = .87), Chile (RMSEA = .090 [CI 90% .086–.095]; CFI = .86; TLI = .85), Perú (RMSEA = .070 [CI 90% .058–.082]; CFI = .88; TLI = .87), and Mexico (RMSEA = .088 [CI 90% .078–.097]; CFI = .89; TLI = .88). In view of this, a bi-factor model of three related factors (model 4) was proposed, which showed adequate fit indexes to the data in Colombia (RMSEA = .068 [CI 90% .063–.072]; CFI = .92; TLI = .90), Chile (RMSEA = .072 [CI 90% .067–.076]; CFI = .92; TLI = .90), and Mexico (RMSEA = .073 [CI 90% .063–.083]; CFI = .93; TLI = .92). However, in Peru, the bi-factor model presented estimation problems, so it was impossible to evaluate the fit of the model.

In Colombia, the bi-factor indices showed that the general factor presents a high explained common variance (ECV) (.90), evidencing that the general factor explains 90% of the variance of the items. Regarding the specific ECVs, factor 1 (.11) and factor 2 (.09) manage to explain 11% and 9% of the common variance, respectively. It was also evident that most of the items are strongly influenced by the general factor (I-ECV > .85). The average relative parameter bias (ARPB) was equal to .01, indicating that the factor loadings of the bifactor model and the factor loadings of a unidimensional model only differ by 1%, which is within acceptable ranges. The H coefficient was equal to .93, which is evidence of stability in other studies, while the Hs for the specific factors were less than .70, providing evidence in favor of a general factor. This is evidence of the relevance of a two-factor model in this country.

For Chile, the bi-factor indices showed that the general factor presents a high ECV (.84), evidencing that the general factor explains 84% of the variance of the items. Regarding the specific ECVs, factor 1 (.24) and factor 2 (.07) manage to explain 24% and 7% of the common variance, respectively. It was also evident that most of the items are strongly influenced by the general factor (I-ECV > .85). The ARPB was equal to .05, indicating that the factor loadings of the bi-factor model and the factor loadings of a unidimensional model only differ by 5%, which is within acceptable ranges. The H coefficient was equal to .94, which is evidence of stability in other studies, while the Hs for the specific factors were less than .70, providing evidence in favor of a general factor. This is evidence of the relevance of a two-factor model in this country.

According to the results found in the countries, model 4 was chosen for the following psychometric analyses, since it was the model that best fitted the data. It is important to mention that Peru was excluded from the following analyses because no solid evidence was found of the adequate functioning of any of the models proposed in the study.

Table 4 shows that the factor loads of the items with the general factor are significant and high in the countries of Colombia, Chile, and Mexico. It can also be seen that the two specific factors have significant factor loads with most of their items in the three countries.

**Table 4:** Factor weights of the bifactor model items in Colombia, Chile, and Mexico

		Factor weight (λ)																			
		1	4	5	8	10	11	13	15	17	19	2	3	6	7	9	12	14	16	18	20
Colombia	FG	.61	.66	.66	.68	.53	.67	.61	.67	.67	.60	.59	.52	.59	.63	.67	.70	.68	.70	.60	.71
	F1	.33	− .17	− .11	.42	.31	.12	− .09	.19	− .04	− .04	–	–	–	–	–	–	–	–	–	–
	F2	–	–	–	–	–	–	–	–	–	–	.19	− .16	.04	− .08	.30	.18	.09	− .03	− .43	− .19
Chile	FG	.62	.73	.68	.53	.43	.60	.60	.60	.69	.61	.60	.50	.71	.67	.59	.60	.73	.79	.61	.80
	F1	.29	− .08	.12	.57	.42	.57	.13	.44	.04	.30	–	–	–	–	–	–	–	–	–	–
	F2	–	–	–	–	–	–	–	–	–	–	.33	− 01	.26	.09	.04	− .06	− .12	.28	− .07	− .17
Mexico	FG	.70	.79	.65	.66	.65	.69	.58	.65	.74	.60	.68	.61	.78	.76	.62	.58	.77	.83	.73	.80
	F1	.14	− .06	− .03	.42	.35	.48	.07	.33	.08	.15	–	–	–	–	–	–	–	–	–	–
	F2	–	–	–	–	–	–	–	–	–	–	.13	− .03	.12	.06	− .55	− .49	− .09	.01	.24	− .01

F1 = Cohesion, F2 = Adaptability

### Factor invariance by country

Table 5 shows that the factor structure of FACES III showed evidence of being strictly invariant between the countries of Colombia, Chile, and Mexico in the sequence of invariance models proposed: metric (ΔRMSEA = .000), scalar (ΔRMSEA = .008) and strict (ΔRMSEA = .008) invariance.

**Table 5:** Invariance model between Colombia, Chile, and Mexico

Invariance models	χ^2^	df	*p*	SRMR	TLI	CFI	RMSEA [CI 90%]	Δχ^2^	Δdf	*p*	ΔRMSEA
Per country											
Configural	2206.98	450	< .001	.038	.90	.92	.070 [.067–.073]	–	–	–	–
Metric	2492.25	524	< .001	.064	.90	.91	.070 [.068–.073]	303.49	74	< .001	.000
Scalar	3214.51	564	< .001	.074	.88	.88	.078 [.075–.080]	908.52	40	< .001	.008
Strict	4018.67	604	< .001	.085	.86	.85	.086 [.083–.088]	733.37	40	< .001	.008

χ2 Chi square, *df* degrees of freedom, *SRMR* standardized root mean square residual, *TLI* Tucker-Lewis Index, *CFI* Comparative Fit Index, *RMSEA* root mean square error of approximation, *Δχ2* differences in Chi square, *Δdf* differences in degrees of freedom, *ΔRMSEA* change in root mean square error of approximation

### Scale reliability

Table 3 shows that the bi-factor model with two specific factors (model 4) showed adequate levels of reliability in the three countries. In Colombia, the hierarchical omega coefficient was adequate for the overall factor (ω_H_ = .91) and for specific cohesion factors (ω_hs_ = .08) and adaptability (ω_hs_ = .06). Similarly, the general factor and its dimensions present an adequate *H* coefficient. (*H*_HG_ = 93; *H*_hs_ = .35; *H*_hs_ = .32 respectively). In Chile, the hierarchical omega coefficient was appropriate for the general factor (ω_H_ = .88) and for specific cohesion factors (ω_hs_ = .17) and adaptability (ω_hs_ = .04). Similarly, the general factor and its dimensions present an adequate *H* coefficient (*H*_HG_ = 94; *H*_hs_ = .62; *H*_hs_ = .26 respectively). In Mexico, the hierarchical omega coefficient was adequate for the overall factor (ω_H_ = .88) and for specific cohesion factors (ω_hs_ = .13) and adaptability (ω_hs_ = .11). Similarly, the general factor and its dimensions present an adequate *H* coefficient (*H*_HG_ = 95; *H*_hs_ = .45; *H*_hs_ = .46 respectively). All this shows that the scale has adequate reliability indexes in the three countries studied.

## Discussion

The present study evaluated the structure and factorial invariance of the FACES III scale in health students from Chile, Colombia, Peru, and Mexico. The results showed that the original model of two dimensions related to twenty items did not fit the data in the four countries. This result was similar to that reported in other studies carried out in Spain (Jiménez et al., 2017; João Forjaz et al., 2002; Martínez-Pampliega et al., 2011), Malaysia (Cong et al., 2022), Japan (Hasui et al., 2004), Argentina (Schmidt et al., 2010), Mexico (Ponce Rosas et al., 2002), Peru (Bazo-Álvarez et al., 2016), and Chile (Zicavo et al., 2012).

In these studies, several items presented crossed loads, very low factor weight, and representativeness problems for the adaptability dimension. Therefore, several studies eliminated items, added correlated errors between items, shifted items to a different factor, or used orthogonal models (Cong et al., 2022; Hasui et al., 2004; Jiménez et al., 2017; João Forjaz et al., 2002; Ponce Rosas et al., 2002). Other studies have suggested the existence of alternative models such as a three-factor model of related factors (Schmidt et al., 2010; Zicavo et al., 2012). However, these alternative models do not conform to the original approach of the circumplex model of the marital and family system (Olson et al., 2000, 2019). The existence of a general second-order factor model has also been proposed in the scientific literature (Martínez-Pampliega et al., 2011).

The study evaluated the fit of three competing models (unidimensional model, second-order general factor model, and Bi-factor model), which showed that the bi-factor model explains the factor structure of the scale better in three of the four countries (Colombia, Chile, and Mexico). In Peru, the model presented estimation problems, which could be linked to the small sample size (Bader et al., 2022). This new proposal coincides with the theoretical model of the test (Olson et al., 2000), as it maintains the existence of two specific factors: cohesion and adaptability. But it also makes it possible to evaluate the existence of a general factor that directly explains the behavior of the items. Although no other bi-factor models have been found in the scientific literature, the presence of high correlations between the dimensions in the current study (Colombia = .99; Chile = .91; Mexico = .96) and previous studies conducted in Spanish-speaking population (Bazo-Álvarez et al., 2016; Caycho & Castilla, 2020) could indicate the existence of a general factor (Cai, 2015). In addition, in the bi-factor model, the specific factors are modeled orthogonally, since the variance shared among the factors is due to the presence of a general factor (Reise, 2012). Likewise, the orthogonal approach to the dimensions allows us to identify the 25 types of family functioning (Olson et al., 2019). The Cohesion dimension allows us to study how family systems balance the separation and union between family members. On the other hand, the dimension of Adaptability, also called Flexibility, allows us to study how family systems balance stability and change in family life (Olson et al., 2019).

Regarding the scale measurement invariance, the sequence of hierarchical invariance models showed that the bi-factor model fits the data in all restrictive models. Specifically, it was found that the bi-factor model shows configural invariance, which supports the presence of the same general factor and its specific factors in both countries. Therefore, these results suggest that nurses and obstetrics personnel in Colombia, Chile, and Mexico conceptualize family functioning in a similar way (van de Schoot et al., 2012). The metric invariance of the model was also demonstrated for the three countries, i.e., it was shown that the general factor and both specific factors are related to the FACES III items in a similar way in the three groups. This shows strong evidence that participants from Colombia, Chile, and Mexico attribute the same meaning to the latent constructs of FACES III (Schmitt & Kuljanin, 2008; van de Schoot et al., 2012).

In addition, it was found that the bi-factor model presents scalar invariance for the three countries. This result shows that the intercepts are the same for all three groups, i.e., participants who have the same score on the latent construct will obtain the same score on the observed variable regardless of the group to which they belong (Milfont & Fischer, 2010). Finally, the strict invariance of the bi-factor model was demonstrated for the three countries. This suggests that the residuals are equal across the three groups, indicating that FACES III measures family functioning with equivalent measurement error among participants in the three countries. All this is important, as it will allow more reliable comparisons between countries based on the sum of observed scores or the estimation of latent means, thus avoiding method bias when making comparisons (van de Schoot et al., 2012). Taking into account that the social and cultural aspect is closely linked to family functioning (Chung & Gale, 2009), cultural, economic, and educational differences in the three countries could lead to different interpretations of the family functioning items. However, the invariance results show that these factors are not strong enough to have a significant impact, and therefore how participants perceive their family functioning when reading the FACES III items is valid in all three groups. It is important to mention that this is the first study that provides evidence on the factorial invariance of the FACES III scale between Colombia, Chile, and Mexico.

Regarding the reliability of the scale, the study shows strong evidence of the internal consistency of the scale in the three countries. These pieces of evidence guarantee a lower measurement error and a higher accuracy of the scores obtained (McDowell, 2006). Furthermore, these results constitute the first empirical evidence of the scale’s internal consistency, using other robust reliability indicators.

## Limitations

The study is not exempt from several limitations. First, non-probabilistic convenience sampling was used, which limits the generalization of the results to the three countries. In addition, in both groups, there was a higher predominance of women and young participants (< 25 years old). There were also differences in sample size between countries, with Colombia and Chile having larger sample sizes. Therefore, the use of probability sampling techniques and larger and more representative samples for all countries is suggested for future studies. The evaluation of other models of invariance between age groups and gender is also recommended. Second, the study assessed only the internal structure-based validity of FACES III in the countries. Other sources of validity such as content validity and validity based on the relationship with other variables were not examined. Therefore, future studies should include variables linked to family functioning, such as family communication and family life satisfaction (Brajsa-Zganec et al., 2017; H. Lei et al., 2020; Lin & Yi, 2017). Third, the temporal stability of the scale was not evaluated. In addition, the study was not longitudinal, so the invariance analysis does not provide any evidence that the construct family functioning is measured in the same way and with the same metrics across different periods (Liu et al., 2017). Therefore, for future studies, evaluation of the reliability of the scale through test-retest methods is suggested. Fourth, self-report measures were used, where participants’ responses could have been affected by social desirability, generating insufficient or excessive responses in some of the items. Several studies have shown that social desirability is mainly related to measures that evaluate important and sensitive aspects of the person, such as health (Latkin et al., 2017; Vesely & Klöckner, 2020). Therefore, it is suggested that future studies include measures that allow for better control of social desirability, such as a specific scale that measures social desirability (van de Mortel, 2008).

## Conclusions

Despite these limitations, it can be concluded that the FACES III scale shows adequate psychometric performance under a bi-factor model in Colombia, Chile, and Mexico. It is important to mention that in the case of Colombia, no previous studies on the factorial structure of the scale were found, therefore, these results constitute the first empirical evidence of FACES III in this context. On the other hand, the scale shows solid evidence of factor invariance between Colombia, Chile, and Mexico. Theoretical and practical implications can be identified in this regard. At a theoretical level, factor invariance tests in different cultures provide relevant information on the similarity and differences in the understanding of the construct in different countries (Boer et al., 2018). Concerning this, the results of the study show that the differences in FACES III scores between countries can be attributed to real differences in family functioning and not to other characteristics of the scale, such as understanding of the items or familiarity with the response categories. On a practical level, FACES III is a short and easy-to-apply metric, which allows its use in different situations such as initial evaluations or epidemiological surveys. In addition, the scale provides useful information on the levels of cohesion and adaptability allowing a better understanding of family functioning in health personnel, especially in nursing and obstetrics personnel. Finally, having a measure for cross-cultural comparisons of family functioning in nursing and obstetrics personnel in Colombia, Chile, and Mexico can provide useful information for the development of common policies that seek to improve well-being related to family functioning in these countries.

## Data Availability

The datasets generated during and analyzed during the current study are available from the corresponding author on reasonable request.

## Abbreviations

ARPB: Average relative parameter bias
CFA: Confirmatory factor analysis
CFI: Comparative fit index
CI: Confidence interval
df: Degrees of freedom
ECV: Explained common variance
FACES: Family adaptability and the cohesion evaluation scale
I-ECV: Item explained common variance
MGCFA: Multi-group confirmatory factor analysis
MLR: Robust maximum likelihood
RMSEA: Root mean square error of approximation
SRMR: Standardized root mean square residual
TLI: Tucker-Lewis Index
